# Recontacting non-BRCA1/2 breast cancer patients for germline *CHEK2* c.1100del pathogenic variant testing: uptake and patient experiences

**DOI:** 10.1186/s13053-021-00166-1

**Published:** 2021-01-19

**Authors:** Mary E. Velthuizen, Rob B. van der Luijt, Beja J. de Vries, Marco J. Koudijs, Eveline M. A. Bleiker, Margreet G. E. M. Ausems

**Affiliations:** 1 Division Laboratories, Pharmacy and Biomedical Genetics, Department of Genetics, University Medical Center Utrecht, P.O. Box 85500, 3508 GA Utrecht, The Netherlands; 2 Department of Clinical Genetics, Leiden University Medical Center, Leiden, The Netherlands; 3 Division of Psychosocial Research and Epidemiology, Netherlands Cancer Institute, Amsterdam, The Netherlands; 4 Family Cancer Clinic, The Netherlands Cancer Institute, Amsterdam, The Netherlands

**Keywords:** Uptake testing, *CHEK2* c.1100del pathogenic variant, Breast cancer risk, Recontacting

## Abstract

**Background:**

*CHEK2* has been recognized as a breast cancer risk gene with moderate effect. Women who have previously tested negative for a *BRCA1/2* gene germline pathogenic variant may benefit from additional genetic testing for the *CHEK2* c.1100del pathogenic variant. The aims of this study were: 1) to assess the uptake of an active approach by recontacting *BRCA1/2*-negative women for additional *CHEK2* c.1100del testing on stored DNA-samples and 2) to explore patients’ experiences with this approach.

**Methods:**

Between 2015 and 2017, women who had been tested earlier negative for *BRCA1/2* germline pathogenic variants, were recontacted for additional *CHEK2* c.1100del testing on stored DNA-samples, free-of-charge. They received an information letter about the *CHEK2* pathogenic variant and could return an informed consent form when they opted for additional genetic testing. Those in whom the *CHEK2* pathogenic variant was absent, received a letter describing this result. Those who tested positive, were invited for a personal counseling at the department of genetics. On average 21 months (range 4–27) after the genetic test result, a questionnaire was sent to all identified carriers and a control group of women who tested negative for the pathogenic variant to explore patients’ experiences with our approach.

**Results:**

In total, 70% (*N* = 1666) of the *N* = 2377 women contacted opted for additional testing, and 66 (4%) of them proved to be carriers of the *CHEK2* c.1100del pathogenic variant. Regardless of the outcome of the genetic test, women were generally satisfied with our approach and reported that the written information was sufficient to make an informed decision about the additional *CHEK2* testing.

**Conclusions:**

The uptake (70%) of our approach was considered satisfactory. Patients considered the benefits more important than the psychosocial burden. Given the rapid developments in DNA-diagnostics, our findings may support future initiatives to recontact patients about additional genetic testing when they previously tested negative for a pathogenic variant in a breast cancer gene.

## Background

Approximately 5–10% of the breast cancers follows an autosomal dominant inheritance pattern and is characterized as hereditary. The high-risk breast cancer susceptibility genes *BRCA1* and *BRCA2* account for up to 30% of these hereditary cases, which leaves a large proportion of familial clustering unexplained [1]. Other breast cancer predisposing genes have been identified including *ATM, CHEK2, PALB2, PTEN, TP53* and *CDH1* [2–6].

The founder germline pathogenic variant *CHEK2* c.1100del (hereafter referred to as *CHEK2* PV) is present in approximately 0.2 to 1.6% of individuals of Northern and Eastern European descent [4, 7, 8]. In the Netherlands, this PV is present in 1.1% of the general population and in approximately 5% of breast cancer cases with a family history of breast cancer [3]. It has been shown that *CHEK2* heterozygotes with a family history of breast cancer have a two- to threefold increased risk of breast cancer [3, 9–11], classifying it as a moderate risk variant. In addition, the risk of contralateral breast cancer is more than two times as high in patients with the *CHEK2* PV compared to patients without this PV [12]. Homozygosity for the *CHEK2* PV is rare, but seems to be associated with a higher breast cancer risk [13, 14].

As of September 2014, routine genetic testing for newly referred breast cancer patients in the Netherlands includes testing for the *CHEK2* PV [14]. Based on the current criteria, women are also eligible for additional *CHEK2* testing if they had previously undergone *BRCA1/2* testing and no PV was detected.

Based on the clinical implications for patients and family members with the *CHEK2* PV and the rather high prevalence in the patient population, it was considered good clinical care to offer additional *CHEK2* testing to women counseled before September 2014, with a negative *BRCA1/2* test result. As we expect more cancer-predisposing genes to be discovered in the near future, our experience with active recontact may be increasingly important for patients with breast cancer who earlier tested negative for PVs in known cancer genes.

The aims of the study were (1) to assess the uptake of recontacting *BRCA1/2*-negative women for additional *CHEK2* PV testing, and (2) to explore patients’ experiences with these approach.

## Methods

### Participants

**Fig. 1:**
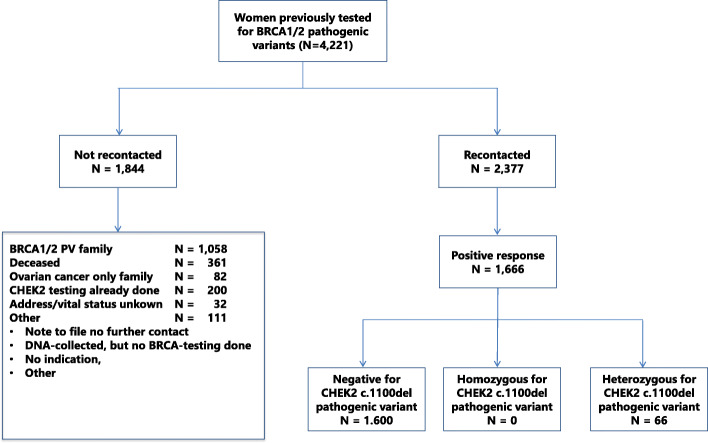
Flow chart study population

Women counseled at the genetics department of the University Medical Center Utrecht (UMC Utrecht) in the period from 1999 to 2014 who met the following criteria were included: 1) a personal or family history of breast cancer and 2) no *BRCA1* or *BRCA2* PV was detected. We excluded counselees who tested negative for a *BRCA1* or *BRCA2* PV that was identified in their family and counselees from ovarian-cancer-only families. A total of 2377 women with a negative test result for *BRCA1* and *BRCA2* gene PVs were eligible for recontacting (Fig. 1).

### Design

Eligible women were identified through our electronic patients records, and the vital status and current addresses were checked. Over a period of 2 years, from November 2015 to October 2017, a letter with information about the *CHEK2* c.1100del PV was sent to all eligible women offering them additional testing for this PV. This letter included information about the possible consequences of an abnormal test result for the patient herself (a higher risk for a second breast cancer and subsequent appropriate screening recommendations) and for her relatives (who may be a carrier of the PV and therefore be eligible for additional screening). Information on possible other cancer risks was not included in the letter. A woman consenting to additional *CHEK2* testing could then return an informed consent form and a reply form stating that she opted for genetic testing for this PV. No new blood sampling was required for the vast majority of counselees because stored DNA-samples collected for the earlier *BRCA1/2* testing were still available. The PV was tested free-of-charge for the counselee using Sanger sequencing in the ISO15189 accredited laboratory for genome diagnostics of the UMC Utrecht. If the PV was absent, a letter describing the test result was sent to the counselee. This letter included a recommendation to contact the genetics department when new cancers were diagnosed in the patient or her relatives in order to adapt the breast cancer risk estimation that was previously discussed with the patient. It also included the recommendation to recontact the department in a few years to inform about additional genetic panel testing. If the *CHEK2* PV was present, the counselee was invited to the genetics department for disclosure of the result and an explanation of the consequences of this finding for the counselee (e.g. increased risk to develop (a second) breast cancer) and her female family members. Counselees were also informed about the breast screening recommendations according to the national guidelines [14]. They were also informed that there were no indications for an increased risk to develop ovarian cancer. A family letter was provided to the counselee to be used to inform her relatives about the findings.

### Study measures

We used a questionnaire to investigate how people responded to our approach, evaluate its psychosocial impact and examine whether *CHEK2* PV carriers experienced our recontact approach differently than a control group consisting of women with a negative test result. This questionnaire was sent out after all tested patients received their genetic test result. For each PV carrier, we selected at least two controls, matched on counseling year and current age. The questionnaire addressed the sociodemographics of the patients (age, education, country of origin), their health status, the psychosocial impact of our approach (anxiety and cancer worries for themselves and/or their family members), the consequences of the genetic test result, and overall satisfaction with the offer for additional DNA testing of the *CHEK2* gene and the decision made by the counselee. These questions were study-specific adapted from validated questionnaires (the Cancer Worry Scale [15] and the Satisfaction with Decision Scale [16]) (Table 3).

Educational level was determined by the Dutch Standard Classification of Education [17] and the international classification of UNESCO [18].

### Statistical analysis

Descriptive statistics were used to describe the baseline characteristics of the PV carriers. To investigate the effect of recontacting, univariate analyses were performed to compare the differences in outcome measures between tested PV carriers and non-carriers. For continuous variables, the independent t-test or the non-parametric Mann Whitney test was used, and for categorical variables, the Chi-Square test or Fisher exact test to address the research questions. All tests were two-sided with a significance level (α) of 0.05. All statistical analyses were performed using SPSS Statistics version 25, IBM SPSS Statistics Corp., Armonk, NY, USA.

## Results

Of the 2377 women recontacted, 70% (*N* = 1666) responded positively to our invitation to have additional testing done for the *CHEK2* PV. In total, 66 women (almost 4%) from 64 families were found to be heterozygous for this PV. No women homozygous for the PV were found. The 1600 proven non-carriers received their test result in writing. The baseline characteristics of the *CHEK2* PV carriers are described in Table 1.

**Table 1:** Characteristics of the *CHEK2* c.1100del index PV carriers (*N* = 66)

Breast cancer	N	Mean age diagnosis	Min	max
Breast cancer	64	44.8	21	64
2nd breast cancer	18	52.1	35	67
Other cancer	7	54.2	39	66
Ovarian cancer (borderline)	3 (2)	50.3	39	65
Skin (1 melanoma; 3 undefined)	4	58	53	66
**Receptor status**	**Pos (%)**	**Neg. (%)**	**Some amplifaction (%)**	
Oesterogen (*N* = 48)	44 (91.7%)	4 (8.3%)		
Progesteron (*N* = 47)	37 (78.7%)	10 (21.3%)		
HER2neu amplification (*N* = 35)	9 (25.7%)	24 (68.6%)	2 (5.7%)	
	**Ductal**	**Lobular**	**Ductal with lobular features**	
Histopathology known (*N* = 53)	45 (84.9%)	5 (9.4%)	3 (5.7%)	
**Family history**	**Yes (%)**	**No (%)**	**Unknown (%)**	
Breast cancer	51 (77.3%)	14 (21.2%)	1 (1.5%)	
1st-degree only	23			
2nd-degree only	14			
Both 1st- and 2nd-degree	13			
Families with other cancers in 1st degree relatives	32 (48.5%)	33 (50.0%)	1 (1.5%)	
N Cancers in 1st-degree relatives^a^	40 (60.6%)			

^a^Colorectal (*N* = 8), prostate (*N*= 5), ovary (*N* = 4), endometrium (*N* = 3), kidney (*N* = 3), skin (*N* = 4, incl. two melanomas), Leukemia (*N* = 2), cervix (*N* = 2), other cancers (*N* = 8)

Almost all PV carriers had been diagnosed with breast cancer (64 out of 66), with a mean age at diagnosis of 49 years. In the breast cancer patients with the *CHEK*2 PV in our study, 91.7% had estrogen-receptor-positive cancer, 78.8% had progesterone-receptor-positive cancer and 68.8% had a negative epidermal growth factor receptor 2 (HER2)-status.

Although ovarian-cancer-only families were excluded from our study, three of 66 index PV carriers were diagnosed with either ovarian cancer or borderline ovarian tumor. Two of these women were also diagnosed with breast cancer.

The majority of the index PV carriers (77.3%) had first- and/or second-degree family members with breast cancer, and 60.6% had at least one first-degree family member with another malignancy besides breast cancer, such as colorectal, prostate, ovarian and endometrial cancer (see Table 1).

All 66 *CHEK2* PV carriers received their test result in-person at the genetics department and were counseled about breast cancer risk, surveillance strategies and the implications for their family members. After the counseling, a family letter was provided to facilitate disclosure of the test result within the family.

To evaluate the patients’ experiences of our approach, a questionnaire was sent to all *CHEK2* PV carriers (cases) who were alive (*N* = 65) and an age-matched control group consisting of women who tested negative for this PV (*N* = 160) several months after receipt of the genetic test result (median 22, range 4–27). A total of 156 women (69%) returned the questionnaire; 52 cases (80%) and 104 controls (65%) on average 21 months after receiving the genetic test results (Table 2). The educational level of responding cases and controls did not differ significantly.

**Table 2:** Characteristics respondents questionnaire

	*CHEK2* c.1100del PV		No *CHEK2* c.1100del PV detected		Total
	***N*** = 52 (33.3%)		***N*** = 104 (66.7%)		
Mean age at time questionnaire (min-max)	59.40 (43–78)		59.75 (31–84)		59.63 (31–84)
Country of origin (1 missing value)					
Netherlands	52		100		152
Other (Indonesia, Israël, Marocco)	0		3		3
Mean time gap test result and questionnaire	19 months		22 months		21 months
Median (min – max)	21 (4–26)		23 (10–27)		22 (4–27)
	**N**	**%**	**N**	**%**	*p*-value
Education					
Low	4	7.7%	16	15.7%	n.s.
Intermediate 1	19	36.5%	23	22.5%	
Intermediate 2	16	30.8%	27	26.5%	
High	13	25.0%	36	35.3%	
No breast cancer	3		5		
Breast cancer	49		99		
Unilateral	37	75.5%	84	84.8%	n.s.
Bilateral	12	24.5%	15	15.2%	
Perceived health					
Excellent/good	45	86.5%	85	81.7%	
Moderate	6	25.0%	18	17.3%	n.s.
Bad	1	1.9%	1	1.0%	
Childless	8	15.4%	12	11.5%	n.s.
Offspring	44	84.6%	92	88.5%	
N daughters	57		106		
N sons	41		101		

Most respondents had children (84.6% of cases and 88.5% of controls). *CHEK2* PV carriers were more often diagnosed with bilateral breast cancer than non-carriers (24.5% versus 15.2%), but the difference was not statistically significant (see Table 2).

Regardless of the results of the PV testing, almost all participants appreciated being recontacted for additional testing (98.1% of PV carriers and 95.2% of non-carriers) (Table 3). More counselees in the PV group (11.5%) tended to experience feelings of anxiety compared to those of the control group (5.8%), and recontacting was more frequently related to concerns about cancer in the family (15.4% for PV carriers versus 1.9% for non-carriers, *p* < 0.001).

**Table 3:** Questions regarding the experiences with recontacting for the additional *CHEK2* mutation test

	*CHEK2* c.1100del PV		No *CHEK2* c.1100del PV detected		Total
	***N*** = 52		***N*** = 104		
**Questions regarding recontacting**	**N**	**%**	**N**	**%**	*p*-value
Recontact appreciated					
agree	51	98.1%	99	95.2%	n.s.
neutral	1	1.9%	5	4.8%	
disagree	0	0.0%	0	0.0%	
Recontacting caused a lot of anxiety for myself					
agree	6	11.5%	6	5.8%	n.s.
neutral	12	23.1%	12	11.7%	
disagree	34	65.4%	85	82.5%	
Recontacting increased my worries for the family					
agree	8	15.4%	2	1.9%	< 0.001
neutral	17	32.7%	4	3.9%	
disagree	27	51.9%	97	94.2%	
Written information sufficient for informed decision					
agree	50	96.2%	99	96.1%	n.s.
neutral	1	1.9%	3	2.9%	
disagree	1	1.9%	1	1.0%	
The decision for additional *CHEK2* genetic testing was difficult					
agree	2	3.8%	2	1.9%	n.s.
neutral	3	5.8%	7	6.7%	
disagree	47	90.4%	95	91.3%	
Difficult to determine advantages and disadvantages of additional testing					
agree	5	9.6%	8	7.7%	n.s.
neutral	7	13.5%	20	19.2%	
disagree	40	76.9%	76	73.1%	
**After receiving the** ***CHEK2*** **test results**					*p*-value
The results of the test were as expected					
agree	15	28.8%	39	39.0%	0.004
neutral	19	36.5%	49	49.0%	
disagree	18	34.6%	12	12.0%	
In retrospect I felt satisfied with having the choice for *CHEK2* PV testing					
agree	51	98.1%	97	96.0%	n.s.
neutral	1	1.9%	4	4.0%	
disagree	0	0.0%	0	0.0%	
The test results influenced cancer worries counselee					
no change in cancer worries	35	67.3%	89	88.1%	< 0.001
yes, increase of cancer worries	14	26.9%	1	1.0%	
yes, decrease of cancer worries	3	5.8%	11	10.9%	
The test results influenced cancer worries family members					
no change in cancer worries	22	42.3%	81	80.2%	< 0.001
yes, increase of cancer worries	27	51.9%	0	0.0%	
yes, decrease of cancer worries	3	5.8%	20	19.8%	
Regrets about the choice for additional genetic testing					
disagree	52	100.0%	103	99.0%	n.s.
neutral	0	0.0%	1	1.0%	
agree	0	0.0%	0	0.0%	
Satisfaction with recontacting					
good	51	98.1%	103	99.0%	n.s.
fair	1	1.9%	1	1.0%	
poor	0	0.0%	0	0.0%	
Satisfaction with information prior to DNA-test					
good	46	88.5%	97	97.1%	n.s.
fair	4	7.7%	3	2.9%	
poor	2	3.8%	0	0.0%	
Satisfaction with information after test results					
good	49	94.2%	97	93.3%	n.s.
fair	3	5.8%	7	6.7%	
poor	0	0.0%	0	0.0%	

Almost all women reported that the written information was sufficient to make an informed decision about the additional *CHEK2* testing (96.2% of the PV carriers and 96.1% of the non-carriers), and slightly less PV carriers were satisfied with the written information that was provided prior to the DNA-test (88.5% of the PV carriers and 97.1% of the non-carriers).

A significant difference was found in the expected outcome of the DNA-test between PV carriers and non-carriers: 34.6% of the PV carriers did not expect the outcome of the test result versus 12.0% of the non-carriers (*p* = 0.004). After disclosure of the test results, the carriers had significantly more cancer worries than the controls, not only for themselves (26.9% carriers versus 1.0% controls, *p* < 0.001), but also for their family members (51.9% versus 0.0%, *p* < 0.001).

## Discussion

To our knowledge, this study includes the largest group of patients recontacted for additional PV testing on newly identified cancer predisposition genes reported so far. As the genetics department of the UMC Utrecht had in the past suggested in the results letter to counselees with a negative *BRCA1/2* test that further genetic testing would be done if deemed possible, we felt an obligation to actively recontact these women. This decision was also made in view of the frequent occurrence of the *CHEK2* c.1100del PV in the Dutch population and the possible clinical implications.

With our approach we identified 66 carriers of the* CHEK2* PV, of whom 64 had been previously diagnosed with breast cancer at a relatively young age (mean age at diagnosis was 49 years). For reference, the mean age of breast cancer diagnosis in the general population in the Netherlands is 61 years [19]. For proven female *BRCA1* and *BRCA2* PV carriers in the Netherlands, the mean ages of diagnosis are 40 years and 44 years respectively [20]. Other clinical characteristics (receptor status) of the identified PV carriers are in line with previous finding [21, 22].

Although the advantages and disadvantages of recontacting counselees for additional genetic tests have been described earlier, there are no recommendations or policies as of yet [23, 24]. In general, there is a lack of consensus about when and whether a genetic counselor has a duty to recontact patients upon the availability of tests for newly discovered genetic PVs [23–25]. Practical obstacles to recontacting are the feasibility of such an effort, including out-of-date contact information and limited resources, both in capacity and financially. On the other hand, recontacting patients may have important implications for these patients and/or their family members regarding their health, lifestyle choices and psychosocial well-being [26].

When considering recontact, we had to take into account that genetic counselling of patients and family members from *CHEK2* PV families is challenging for several reasons. The risk estimates published thus far are based on limited data and might not be very accurate yet. Furthermore, for counseling purposes relative risks have to be translated into absolute risks and more age-specific risk estimates are needed but not yet available [5]. For instance, it has been suggested that the breast cancer rate ratio declines with age for this PV [3]. Also, increased risks for other malignancies besides breast cancer are reported as part of the tumor spectrum [27–30]. As *CHEK2* is now part of a multigene panel for testing of breast cancer patients, more accurate risk estimates for breast cancer and other phenotypic information will become available in the near future. In the Netherlands a nationwide study recently started that will address the risk prediction, screening and therapy of breast cancer in women from *CHEK2* c.1100del families. This study is part of the Hereditary Breast and Ovarian cancer study Netherlands [31].

The uptake of genetic testing in our recontacted counselees was higher than that earlier reported by Romero et al., who recontacted patients with medullary thyroid carcinoma and pheochromocytoma or paraganglioma for additional genetic testing [32]. Chadwell et al. have reported that, in general, the cost of testing and insurance coverage might be the most important barriers to additional genetic testing [33]. In our study the genetic test was done free-of-charge and the visit to the genetics department for the PV carriers was covered by the participant’s health insurance. Our high response rate may also be due to the fact that it took relatively little effort for participants to return the informed consent form and the reply form stating that they opted for additional PV testing. Even the drawing of blood was not necessary because DNA-material was still available.

There was a concern about the potential negative psychosocial consequences of our approach as women may not want to be reminded of their illness and of the consequences the test results might have for herself and her family members [23, 25, 34]. Giesbertz et al. (2019) suggested that practical guidelines are needed to weigh the arguments in favor or against recontacting [23]. Our findings suggest that the women recontacted for additional genetic testing appreciated our effort and that the written information was sufficient to make an informed decision about the additional *CHEK2* testing regardless of the test result and the possible anxiety it caused. This is an important finding in the light of the rapid developments within DNA-diagnostics, as it is conceivable that additional genetic testing on newly identified cancer predisposing genes will be offered more often in future.

An important limitation in our study is that the questionnaire on the impact of our approach comprises only a small sample (*n* = 104) of the 1600 non-carriers opting for the additional test. Furthermore, we do not have survey data on counselees who declined additional genetic testing. These women might have experienced our recontacting differently and perhaps had serious objections to an additional genetic test. A major limitation is that the questionnaire was sent after all additional *CHEK2* testing was finished. Since it took us 2 years to recontact and test all eligible women there was a gap (on average 21 months) for some participants between the receipt of the test results and the completion of the questionnaire, and this might have affected the answers to our questions.

For the most part, the counselees who opted for additional *CHEK2* testing considered the written information provided sufficient to decide about genetic testing. Our findings give an important message to health care professionals, such as gynecologists, medical oncologists and breast surgeons, as they will increasingly deliver treatment-focused genetic testing as part of mainstream breast cancer care [35].

## Conclusions

In this study we assessed the uptake and patients’ experiences of recontacting a large number of women who had previously tested negative for *BRCA1/2* PVs for additional *CHEK2* testing. The uptake of our offer to perform *CHEK2* testing was high (70%), and the PV detection of 4% was in line with previous findings [3, 10]. No patients with a homozygous *CHEK2* PV were found.

Overall the women who filled out the questionnaire were positive about the effort taken to approach them for additional testing for the *CHEK2* PV. Despite the fact that the PV carriers more often experienced cancer worries and anxiety after the test result was revealed, the benefits of our approach seemed to outweigh the psychosocial burden. In fact, eight respondents specifically stated in the comments box at the end of the questionnaire, that they would like this approach to be a routine course of action in future.

## Data Availability

The datasets used and/or analysed during the current study are available from the corresponding author on reasonable request.
